# Ulcers of the Mouth

**Published:** 1868-03

**Authors:** Henry S. Chase


### ARTICLE VII.

### *Ulcers of the Mouth.*

A paper read before the St. Louis Dental Society, by

Henry S. Chase, D.D.S.

The ulcers found in the mouth are various in their appearance and pathology, and still more various in their origin. Ulcers of the mouth or any other part of the body are seldom idiopathic, but *rather* symptomatic. That is, they are rarely local in their origin, but are, on the other hand, only symptoms of some general dyscrasia, or of some local disease in another part of the body.

Young practitioners of Surgery are too apt to limit their view to the object before them, instead of looking for the forces which produced and sustain that object. The true medical philosopher must embrace in his investigations all those elements which act as *causes* for the production of those diseased conditions which he is called upon to relieve and cure.

If then the cause of an ulcer or ulcers in the mouth is not localized in the mouth itself, we must push our inquiries and investigations in that direction which will reveal it. To this end we must not only form our judgment from present symptoms, but we must, by inquiries from the patient, elicit the information we desire. And right here we see that the *medically* educated dental practitioner has a vast advantage over one who has not acquired such knowledge. The latter is groping in the dark among such inquiries, while the former enjoys the delightful sunlight of scientific knowledge, and focalises this light whenever and wherever he pleases, for the gratification of his own scientific tastes and for the good of the world around.

If the dentist is not often called upon to treat a great variety of ulcers of the mouth, it is for two very good reasons, namely: first the *patient*, thinking him ignorant of medical knowledge, does not apply to him for relief; secondly, most of the ulcers of the mouth are concomitant with some other disease, which is being treated by the general practitioner.

Notwithstanding these facts let us study the subject, and make ourselves somewhat familiar with it, for our responsibilities are not in the least lessened by the ignorance of the public, or by that of others in our profession.

What is an Ulcer? It is a disorganized portion of either the external or internal skin, involving more or less of the loose cellular tissue beneath it; having also a circumscribed size and shape, dependent on its pathological character.

We have ulcers in the mouth resulting from syphilis, mercurial poisoning, stomatitis, typhoid fever, typhus fever, intermittent fever, scarlet fever, scrofula, bilious fever, dyspepsia, etc., etc; also, the nursing sore mouth of mothers or nurses.

In addition to mercurial poisoning, there is also that produced by arsenic, nit. acid, mur. acid, sulph. acid, and other drugs. In scorbutic diseases there are also ulcers of the mouth. Now the treatment of these ulcers must accord with that of the disease to which it is correlated. The treatment of an ulcer of the mouth resulting from syphilis, must be the same as that of syphilis itself; and so too in regard to every affection which I have named. Therefore I will leave the study of most of these ulcers for the time when the original diseases themselves shall be considered, and for the present only speak of that more common condition of the mouth which we are called upon much oftener to treat.

### STOMATITIS MATERNA.

This is a disease peculiar to women during the periods of gestation and lactation. It is a constitutional disease, having its origin nearly always in mal-nutrition. There is a greater call on the stomach for the ingestion of nutritious food than in ordinary conditions; for the blood must now furnish to the various tissues of the new being those elements of which it is to be composed, and the food itself must contain those elements in abundance or otherwise, the mother must furnish them at the expense of her own tissues. In the latter case there is *starvation* to a certain degree, and a similar state of the body ensues to that which is found in ship scurvy. Starvation takes place when any *one* of the important elements of the body is denied, as surely as though all of them are refused. In the disease of which I am speaking it is the phosphate of lime which the new being so loudly calls for, as it must be had, not only to form the bones and teeth, but also every other tissue and organ in the body. Flour eaters, or at least those who live principally on food made from the fine flour of wheat are particularly subject to this complaint; for the very good reason that there is scarcely any of the phosphate of lime in fine flour, it being nearly all left in the bran and coarse portions which are sold for the feeding of cattle!

The first symptoms are a feeling of weariness, not only of the muscles, but of the brain; the latter becomes dull, wanting in activity, and desponding; there is a loss of weight in all the tissues; the blood becomes anaemic, or there is leukemia; the tongue is light colored, and the mucous membrane of the lips and mouth is pale and pasty; the teeth leave indentations in the sides of the tongue; the latter often becomes covered with pale, small ulcers, which sometimes coalesce so as to become large patches, and the same occurs also on the gums and cheeks. This pathological condition may be seen far down the throat, and doubtless does obtain in the stomach. There is a loss of appetite; there may be either constipation or diarrhea—the latter is the more common; there is sometimes severe, nervous headache—but oftener a dull and oppressive pain; accidental wounds heal with difficulty; pus cells are proliferated at the expense of the tissues; the breath is often sour; eructations from the stomach the same; the saliva has an acid reaction, and the teeth have an obstinate tendency to decay.

In the severest cases catarrhal symptoms come on, and there is a discharge from the bronchi of the lungs; and as exhaustion proceeds, the patient finally succumbs to literal starvation.

If a child is nursed by the mother it is not independent of the pathological influences affecting the latter; if it does not have apthæ itself it will be very likely to suffer from cholera infantum in the summer. In very many cases it ceases growing and becomes affected with rachitis. If it should live to erupt teeth they will be found to be very imperfectly calcified.

This is a terrible disease, and the more so because so insidious. The mouth trouble or stomatitis is only a symptom of the real disease.

It may be said that I have *overdrawn* the picture; I have only told the truth. But *every* case of stomatitis materna is not the result of impoverished blood, but may be the result of temporary indigestion; or of an irritation of the stomach produced by some article of food disagreeing with that organ, or else the effect of some medicinal substance taken purposely or by accident.

Now if it is true that this is a constitutional disease we must see that *local* remedies will fail. They may, and often do, relieve the mouth symptoms, and under their influence the ulcers may be temporarily cured, but they should be used in connection with those general remedies which will act on the whole system. It will be seen from *my* point of view that hygienic measures must be made very prominent in the treatment.

There should be a *nourishing diet* immediately prescribed, consisting of plain and simply cooked food which has not been robbed of any of its elements of nutrition. At the risk of being tedious I would name unbolted wheat meal, lean beaf and mutton, garden roots, fruits, etc.; also, light native wines and lager beer. I would prohibit fine flour, pork, salted meats, smoked fish, corn starch, pastry and confections, excepting simple fruit jellies, unspiced. There should be moderate exercise, fresh air, sunlight in abundance, daily tepid baths, and any other common sense hygienic measures that may suggest themselves.

Medical treatment may consist in the exhibition of small doses of mercury, quinine, rhubarb, nitric, muriatic, and sulphuric acids, or of citric acid. Arsenic also in small doses is very beneficial. Only *one* medicine at a time should be given, and it should be continued at least a week without change, if we would observe its effects. I think the acids will be found most beneficial where there have been mal-nutrition and starvation; mercury, quinine, and rhubarb, most useful in recent cases of gastric irritation; and arsenic in cases of great debility and prostration, especially when accompanied by catarrh.—*St. Louis Med. Reporter*.

